# Harnessing hyperthermostable lactonase from *Sulfolobus solfataricus* for biotechnological applications

**DOI:** 10.1038/srep37780

**Published:** 2016-11-23

**Authors:** Benjamin Rémy, Laure Plener, Laetitia Poirier, Mikael Elias, David Daudé, Eric Chabrière

**Affiliations:** 1 Aix Marseille Univ, INSERM, CNRS, IRD, URMITE, Marseille, France; 2 Gene&GreenTK, Faculté de Médecine, 27 boulevard Jean Moulin, 13385 Marseille Cedex 5, France; 3 University of Minnesota, Department of Biochemistry, Molecular Biology and Biophysics & Biotechnology Institute, St. Paul, MN 55108, USA

## Abstract

Extremozymes have gained considerable interest as they could meet industrial requirements. Among these, *Sso*Pox is a hyperthermostable enzyme isolated from the archaeon *Sulfolobus solfataricus*. This enzyme is a lactonase catalyzing the hydrolysis of acyl-homoserine lactones; these molecules are involved in Gram-negative bacterial communication referred to as quorum sensing. *Sso*Pox exhibits promiscuous phosphotriesterase activity for the degradation of organophosphorous chemicals including insecticides and chemical warfare agents. Owing to its bi-functional catalytic abilities as well as its intrinsic stability, *Sso*Pox is appealing for many applications, having potential uses in the agriculture, defense, food and health industries. Here we investigate the biotechnological properties of the mutant *Sso*Pox-W263I, a variant with increased lactonase and phosphotriesterase activities. We tested enzyme resistance against diverse process-like and operating conditions such as heat resistance, contact with organic solvents, sterilization, storage and immobilization. Bacterial secreted materials from both Gram-negative and positive bacteria were harmless on *Sso*Pox-W263I activity and could reactivate heat-inactivated enzyme. *Sso*Pox showed resistance to harsh conditions demonstrating that it is an extremely attractive enzyme for many applications. Finally, the potential of *Sso*Pox-W263I to be active at subzero temperature is highlighted and discussed in regards to the common idea that hyperthermophile enzymes are nearly inactive at low temperatures.

Since the emergence of directed evolution and the beginning of the third wave of biocatalysis, enzymes have gained considerable interest for biotechnological purposes^1^. As a consequence, the global market for enzymes is expected to reach US$ 7.1 billion by 2018^2^. However, enzyme use can be limited by cost, activity levels, or incompatibility with existing industrial production plants^3^. Overcoming these limitations has been extensively investigated during the last decade by either stabilizing highly active biocatalysts or isolating enzymes from extreme environments (also known as extremozymes)^4^,^5^,^6^,^7^,^8^,^9^. Psychrophile and thermophile organisms are among those that have been studied in this way^10^,^11^,^12^,^13^. Psychrophilic enzymes have been proven to be efficient at low to moderate temperatures (4–25 °C) and their tolerance to solvents has been highlighted^3^. Conversely, thermophilic enzymes are particularly active at high temperatures (>55 °C) and are known to resist denaturing agents including proteases, surfactants or detergents^3^,^9^. These features increase the compatibility of these biocatalysts with industrial processes and are continuously under consideration for developing efficient enzyme-based technologies.

*Sso*Pox is a phosphotriesterase-like lactonase (PLL) isolated from the archaeon *Sulfolobus solfataricus*^14^. This enzyme is a natural lactonase that is able to hydrolyze acyl-homoserine lactones (AHL) that are involved in the quorum sensing (QS) of Gram-negative bacteria (e.g. *Pseudomonas aeruginosa*, *Acinetobacter baumannii*)^15^,^16^. QS is a communication mechanism by which bacteria sense their population density and synchronize their behavior^17^,^18^,^19^,^20^. Virulence factor secretion and biofilm formation are for example regulated by QS^21^,^22^,^23^. Strategies aiming at counteracting QS, dubbed quorum quenching (QQ), are of prime interest for developing therapeutic alternatives to classical antimicrobial agents (e.g. antibiotics)^24^,^25^,^26^. Industrial applications using QQ strategy mainly include medical devices such as catheters, aerosols or dressings and have been exhaustively reviewed elsewhere^27^,^28^. QQ also appears to be a promising strategy for anti-fouling applications^29^.

In addition to its lactonase activity, *Sso*Pox also displays promiscuous phosphotriesterase activity that can degrade organophosphorus chemicals (OP)^30^. OP are highly toxic compounds that inhibit acetylcholinesterase, a key enzyme for regulation of the central nervous system^31^,^32^. The exhaustive use of OP for agricultural purposes has led to serious contamination worldwide and is a major environmental and public health issue^33^,^34^,^35^. OP were also considered for military ends leading to the synthesis of noxious chemical warfare nerve agents (CWNA)^36^,^37^. These compounds constitute a serious threat for civil and military populations but no satisfactory external decontamination method is currently available^38^. OP-degrading enzymes have thus emerged as potential bioremediation alternatives^38^,^39^.

Considering both its lactonase and phosphotriesterase capabilities as well as its exceptional thermal stability (T_m_ = 106 °C), many applications involving *Sso*Pox are considered^38^,^40^,^41^,^42^,^43^,^44^,^45^,^46^. Although its natural robustness confers outstanding biotechnological potential on *Sso*Pox^47^, its catalytic activities were first increased to turn the biocatalyst into a cost-effective technology^48^. The resolution of the 3D-structure allowed the identification of the crucial role of residue W263 in both activity and substrate promiscuity^15^. Site-saturation mutagenesis was applied and led to the characterization of catalytically improved variants maintaining strong robustness^48^. Among these, the single variant *Sso*Pox-W263I was of special interest insofar as both its AHL- and OP-degrading activities were increased as compared to wild-type enzyme while harboring a high melting temperature value (T_m_ = 88 °C).

Variant *Sso*Pox-W263I, which exhibits higher lactonase and phosphotriesterase activity, is a promising candidate for the external bioremediation of OP, including liquid decontamination solutions, filtration systems, and auto-decontaminating textiles or materials. Additionally, its ability to interfere with bacterial signaling offers a wide variety of possible uses, such as medical devices containing enzymes and biomaterials. However, the industrial feasibility of such bio-based products is dependent on the ability of the variant to meet process requirements. We evaluated the compatibility of the improved variant *Sso*Pox-W263I in harsh conditions. The variant was produced at pilot scale (500 L fermentation) and used for the biotechnological characterization under rough process-like conditions. The variant’s resistance to temperature, solvents, sterilization, bacterial degradation and the enzyme’s lifetime were determined. Our results show that *Sso*Pox-W263I maintains most of its activity after extremely harsh treatments, highlighting the tremendous potential for using this enzyme in existing industrial production lines.

## Results

### Resistance to temperature-induced stress

**Figure 1:**
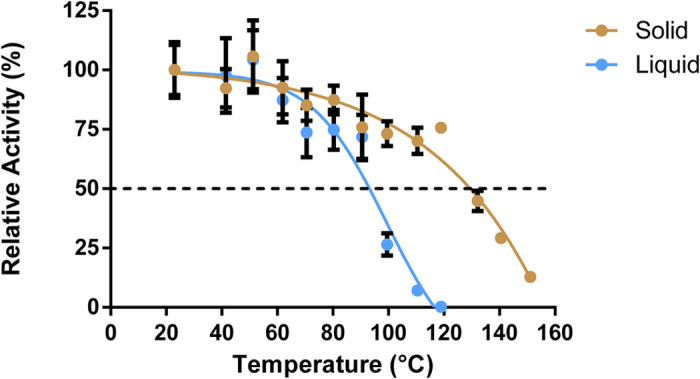
Relative activity of *Sso*Pox-W263I submitted to a 5-minute heat shock in either liquid (blue) or solid form (brown). The values represent the mean ± SEM (Standard Error to the Mean) of six replicates.

Industrial processes often require short but high-temperature steps for various purposes including drying, polymer reticulation, solubilization, chemical reactions^3^,^9^. We investigated the resistance of *Sso*Pox-W263I submitted to a heat shock from 40 °C up to 120 °C in liquid form (*i.e.* atomized powder resuspended in water) and 150 °C in solid form (*i.e.* atomized powder) for 5 minutes using a dry bath to simulate reticulation step used for example in textile processes (Fig. 1). In liquid form, activity only fell slightly up to 90 °C. From 100 to 120 °C, the activity decreased quickly and was almost null at 120 °C. The post-shock activity of the powder form was measured after resuspension in water. Activity only fell marginally with heat shock up to 120 °C. From 130 to 150 °C, activity decreased quickly, albeit more than 10% of the activity remained at 150 °C. The temperatures required to lose one half of enzymatic activity in 5 minutes were found to be 92 °C and 130 °C in liquid and solid states respectively.

### Resistance to sterilization methods

The enzyme was subjected to three sterilization methods: autoclaving, ethylene oxide and β-radiation.

**Figure 2:**
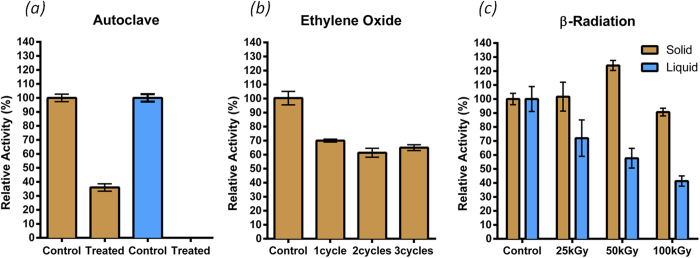
*Sso*Pox-W263I relative activity after submission to three sterilization methods: autoclave (**a**), ethylene oxide (**b**), β-radiation (**c**). Experiments on solid enzyme are in brown bars while those on liquid enzyme are in blue. Measures were performed in triplicate as compared to a non-sterilized control. Values represent means ± SEM.

Autoclaving is commonly used in medical applications for sterilizing materials. It uses a combination of elevated temperature and pressure and is usually destructive for proteins. Here we autoclaved the enzyme in liquid or in solid state (powder) at 121 °C for 15 minutes (Fig. 2a). Surprisingly, more than 30% activity remained after autoclaving the enzyme powder, while all the activity was lost in the case of the liquid form, which is consistent with the temperature resistance of the liquid enzyme.

Chemical gaseous sterilization is used for disinfecting and sterilizing instruments whose properties would be affected in liquid form. We investigated the alkylating agent ethylene oxide for sterilizing the solid enzyme (Fig. 2b). Only 30% of enzyme activity was lost after the first sterilization cycle, which underscores the high robustness of the protein. Interestingly, no additional loss was observed after three repeated sterilization cycles.

The last method we investigated was β-radiation (Fig. 2c). This process is commonly used for high-throughput sterilization with a typical dose of 25 kGy. Here, we considered three distinct doses from 25 kGy up to 100 kGy for sterilizing both liquid and solid forms. With the liquid enzyme, the remaining activity decreased down to 40% at the highest dose. Conversely, the solid form was barely, if at all, affected by radiation. This result is particularly interesting because β-radiation is probably the most convenient sterilization method for medical devices. Radiation may also be used for liquid enzyme sterilization but the dose level may impact the remaining activity.

### Activation by bacterial secretion factors

**Figure 3:**
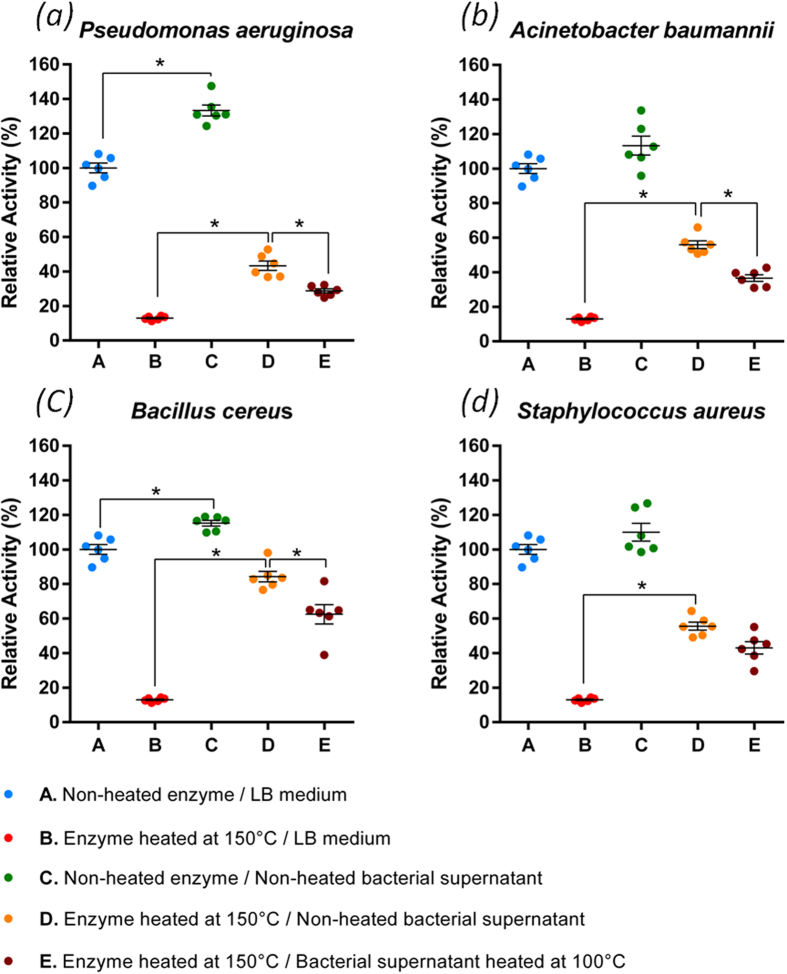
Dot plots of the relative activity of *Sso*Pox-W263I in contact with bacterial culture supernatants of *P. aeruginosa* PAO1 (**a**), *A. baumannii* AYE (**b**), *B. cereus* (**c**) and *S. aureus* (**d**). For each graph, blue dots correspond to the positive control (*i.e.* unheated enzyme resuspended in sterile LB medium). Red dots correspond to the heated control (*i.e.* enzyme heated at 150 °C and resuspended in sterile LB medium). Green dots represent the enhanced activity of unheated enzyme resuspended in bacterial supernatant. Orange dots illustrate the reactivation of heat-shocked enzyme resuspended in bacterial supernatant. Finally, purple dots represent the activity of heated enzyme resuspended in previously 100 °C heated bacterial supernatant to investigate the role of thermolabile compounds in the reactivation phenomenon. The n = 6 replicates are represented with mean ± SEM (Standard Error to the Mean) in black bars. Black stars (*) indicate a significant difference (p < 0.01) according to the corresponding contrast.

We investigated the ability of bacterial secretion materials to promote enzyme activity or reactivate heat-shocked enzyme. Four strains of both Gram-negative and Gram-positive bacteria were considered. Culture supernatants of *Pseudomonas aeruginosa* PAO1, *Acinetobacter baumannii* AYE, *Staphylococcus aureus* ATCC 29213 as well as a clinical isolate of *Bacillus cereus* were investigated for how they affect *Sso*Pox-W263I activity (Fig. 3). Many pathogenic bacteria secrete agents such as proteases or surfactants to inactivate host proteins. Surprisingly, enzyme activity did not decrease in the presence of bacterial supernatant and, in some cases, increased as compared to the LB medium control. Next we considered the ability of supernatants to reactivate heat-shocked enzyme at 150 °C. All the supernatants favored enzyme reactivation, from 40% for *P. aeruginosa*, up to 80% for *B. cereus*. To evaluate the thermo-susceptibility of the reactivation phenomenon, supernatants were further heated to precipitate unstable compounds. Interestingly, the reactivation was less pronounced after heating the supernatant for 10 minutes at 100 °C, underlining the fact that thermolabile metabolites and/or proteins are partially responsible for enzyme reactivation.

### Catalytic activity at sub-zero temperatures

**Figure 4:**
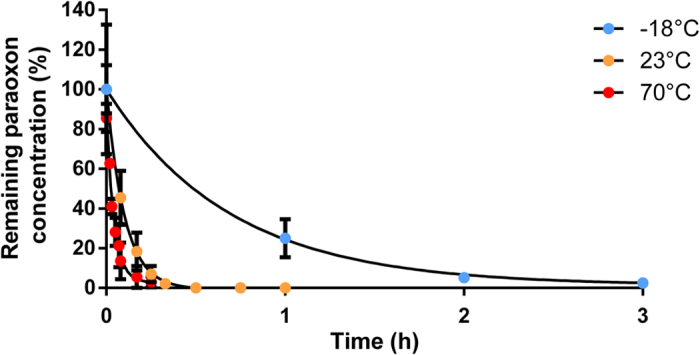
Hydrolysis of paraoxon by variant *Sso*Pox-W263I over time at 70 °C (red dots), 23 °C (orange dots) and −18 °C (blue dots) with 50% glycerol. Relative concentrations of paraoxon are presented. Measures were performed in triplicate and are represented with mean ± SEM in black bars.

Hyperthermostable enzymes are commonly described as nearly inactive at room temperature, but their catalytic power is in fact greater at lower temperature^3^,^49^,^50^. Optimum *Sso*Pox-W263I activity was previously determined in the range of 80–95 °C and significant activity was also observed at 23 °C^14^,^30^,^48^. Here, we investigated the ability of the enzyme to work at sub-zero in glycerol-complemented buffer (Fig. 4). Surprisingly, the variant was still significantly active at −18 °C. The *k*_cat_/K_M_ values were estimated using a low ethyl paraoxon concentration (250 μM) and values of 2.0 × 10^4^ M^−1^.s^−1^, 9.7 × 10^3^ M^−1^.s^−1^ and 1.4 × 10^3^ M^−1^.s^−1^ were determined at 70 °C, 23 °C and −18 °C respectively (Table 1). Rates only decreased by 14-fold and 7-fold at −18 °C, as compared to 70 °C and room temperature, and converted more than 99% of substrate in 6 hours with only 0.3 μM of enzyme. In a previous work, the temperature coefficient (Q_10_) for > 100 enzymatic reactions was found to be 2, including those involving thermostable enzymes^49^. Thus, the average enzymatic rate decrease by ~446-fold and ~17-fold over the same temperature ranges, 70 °C/−18 °C and 23 °C/−18 °C, respectively. The temperature dependence of *Sso*Pox-W263I is therefore much lower than the average enzyme. Consequently, the 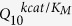 temperature coefficient values calculated between 70 °C/23 °C, 70 °C/−18 °C and 23 °C/−18 °C varying from 1.17 to 1.60 were found to be significantly lower than for most enzymes (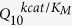 value is ≈2)^49^.

### Tolerance to solvents

**Figure 5:**
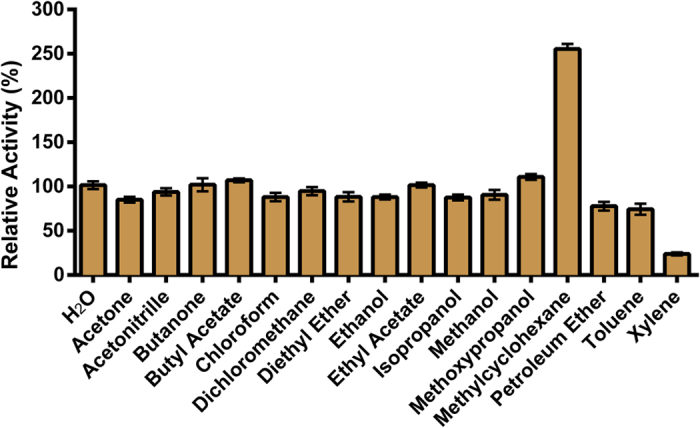
Relative activities of *Sso*Pox-W263I after treatments in different solvents. For each solvent, the n = 6 replicates are represented with mean ± SEM in black bars.

As for temperature, compatibility with solvents is a major prerequisite for processing viability^51^,^52^. Here, we evaluated the potential of *Sso*Pox-W263I to resist to a solvent-requiring industrial step. A broad panel of 16 solvents was considered: acetone, acetonitrile, butanone, butyl acetate, chloroform, dichloromethane, diethyl ether, ethanol, ethyl acetate, isopropanol, methanol, methoxypropanol, methylcyclohexane, petroleum ether, toluene and xylene (Fig. 5). Solid enzyme powder was resuspended in pure (100%) solvent that was further evaporated. The enzyme was subsequently solubilized in water and activity was measured and compared to the water-treated control. Only four out of the 16 solvents investigated significantly affected the activity of *Sso*Pox-W263I (*i.e.* methylcyclohexane, petroleum ether, toluene and xylene). Among these, petroleum ether and toluene only slightly reduced the activity, down to 78% and 74% respectively as compared to the water control. Xylene was the most aggressive solvent as it decreased activity by more than 75%. Conversely, methylcyclohexane surprisingly improved activity by ≈2.5-fold increase (255%). For the 12 others, no significant effect on the enzyme was detected after 2-hour long contact. *Sso*Pox-W263I showed impressive resilience to pure organic solvents.

### Storage capacity of *Sso*Pox-W263I

**Figure 6:**
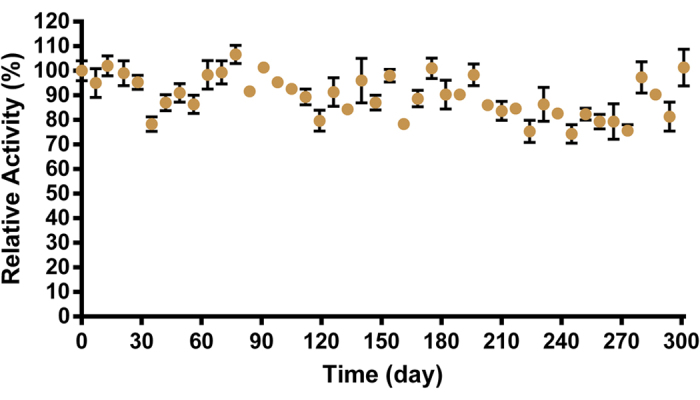
Dot plots of the relative activity of solid enzyme stored at room temperature over time. For each time, the n = 3 replicates are represented with mean ± SEM in black bars.

Hyperthermostable enzymes, with their intrinsic robustness, usually show enhanced lifetime as compared to their mesophilic counterparts. This constitutes a major advantage for long-term storage with minimal constraints. Moreover, lyophilization and atomization usually preserve unstable compounds by minimizing water activity and/or limiting contamination by microorganisms, in addition to being convenient for transport purposes. We followed the activity of solid enzyme stored at room temperature in anhydrous conditions. Interestingly, the enzyme remained active in these experimental conditions for > 9 months. Only a very slight activity decrease can be observed (Fig. 6). This result strongly underlines the industrial relevance of *Sso*Pox-W263I, which may be kept for weeks without alteration.

### Immobilization of *Sso*Pox-W263I

**Figure 7:**
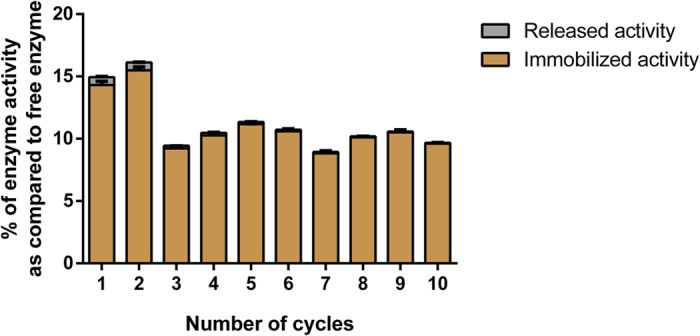
Immobilization rate of enzyme by crosslinking in alginate beads as compared to the free enzyme and recovery. The results for each cycle are represented with mean ± SEM in black bars. Immobilized and released activities are shown in brown and grey respectively.

We investigated the immobilization of *Sso*Pox-W263I through crosslinking aggregation into alginate beads. Glutaraldehyde (GAD) was previously reported as an aggregating agent^53^, and was used here for crosslinking the enzyme inside alginate beads. Only a small amount of enzyme leaked out of the beads during the rinsing steps and the enzyme remained active and accessible after entrapment. Up to 15% of activity was immobilized with this technique. Bead recovery was subsequently investigated and the immobilized enzyme was used ten times with only 30% decrease (Fig. 7). This method offers a simple procedure for the cheap and efficient immobilization of *Sso*Pox-W263I.

## Discussion

Enzymes isolated from extreme environments have gained considerable interest for biotechnological applications. Extremozymes were largely found to be both robust and evolvable, to be optimizable through protein engineering strategies while maintaining tremendous stability. In this work, we first investigated the robustness of a previously engineered *Sso*Pox variant improved against OP and AHL. We show that *Sso*Pox-W263I exhibits remarkable heat resistance, with no drastic effects observed up to 90 °C on liquid or solid samples, which is consistent with the extremophile origin of the enzyme and the Tm value of *Sso*Pox-W263I (88 °C). The enzyme in solid form was found more resistant to temperature as compared to the liquid form, which is probably due to a lower molecular agitation and water activity. Although this extreme robustness is rare for enzymes, other reports have described the tremendous resistance of solvent free protein or super-oxide dismutase which tolerates autoclaving^54^,^55^. Temperature resistance is a major prerequisite for the biotechnological use of enzymes, nevertheless many other aspects have to be considered as well. For example, developing enzyme-based medical devices requires a sterilization step with no or limited loss of activity. We applied three methods, autoclaving, ethylene oxide and β-radiation to sterilize *Sso*Pox-W263I with little or no impact on activity. This result is particularly promising: indeed, contrary to the atomized enzyme, the enzyme incorporated into a medical device would be partially protected by the material from external exposures and would be potentially less affected by the sterilization processes.

Medical devices functionalized with enzymes, such as dressings or catheters, would have to remain active when exposed to bacteria-rich environment such as infected wounds. In this way, we have demonstrated the ability of *Sso*Pox-W263I to resist the molecular arsenal, such as proteases or surfactants, secreted by different bacteria for the degradation of exogenous proteins. The high resistance of the variant to bacterial secretions was observed with both Gram-negative and Gram-positive strains. Enzymatic activity was in fact increased by bacterial culture supernatants. Surprisingly, the supernatants were also able to reactivate the heat-shocked enzyme. Whereas these results match the common observation that hyperstable enzymes are resistant to surfactants or proteases that may be secreted by bacteria, the mechanisms for these two observations are unclear and will require further investigations.

In this study, we demonstrate that *Sso*Pox-W263I is active over an extremely wide range of temperature. Indeed, we have confirmed that the enzyme exhibits very similar catalytic activities at 70 and 23 °C. More surprisingly, we also showed that this enzyme is active at sub-zero temperature and that its temperature dependence is lower than that of most enzymes. Our results underline the fact that extremozymes do not necessarily require high temperatures to be functional and effective. This wide range of activity may be useful for a large panel of applications including bioremediation in permanently cold regions or during winter, the treatment of low-temperature effluents, and anti-fouling in cold seas.

Along with temperature resistance, tolerance to solvent is valuable for biotechnological considerations. However, unlike most extremozymes from psychrophilic origins, hyperthermostable enzymes may be inactivated by solvents. Resuspension of the enzyme in pure organic solvents followed by an evaporation step had only mild effects on the enzyme’s catalytic efficiency. Although the enzyme’s solubilities in organic solvents were not evaluated, these results indicate that solvents may be used as carrier for *Sso*Pox-W263I without altering its efficiency (except for xylene) which is particularly attractive for chemical processes with solvent-required steps or functionalization steps.

Industrial applications also require the enzyme to be compatible with storage. We thus evaluated the activity of *Sso*Pox-W263I over time. We found that the enzyme remained active over months at room temperature with variation in activity in the range of <25%. Finally, we have immobilized the enzyme and tested its activity. Industrial applications using enzymes often require an immobilization step that offers many advantages as compared to the utilization of soluble enzyme. Immobilization usually increases recyclability and stability, while the downstream process is simplified and the contamination risk is reduced. Many strategies may be considered for immobilizing enzymes, including adsorption, covalent binding, entrapment and crosslinking^56^,^57^,^58^. Reaching a compromise between support cost and binding capacity is required to optimize the competitiveness of the product. We have immobilized *Sso*Pox-W263I on alginate beads using glutaraldehyde crosslinking. The immobilized enzyme remained active and very little (if any) enzyme release was observed in the reaction medium. This suggests that such solid biocatalysts may be used for filtration purposes for various applications including the bioremediation of OP for decontaminating soiled effluents.

To conclude, this study demonstrates the exceptional properties of *Ss*oPox-W263I in regards to its resistance to temperature, solvents, proteases, sterilization processes, storage as well as immobilization procedures. These properties strongly support that *Ss*oPox-W263I is manipulable for biotechnological considerations as it matches several industrial requirements. Many applications involving this variant may be envisaged for either OP or AHL degradation, such as filtration systems, anti-fouling paints, medical devices, self-decontaminating materials, and textiles.

## Methods

### Batch characteristics

The production was performed in a 500-L tank and was adapted from a previously reported lab-scale procedure. Briefly, strain *Escherichia coli* BL21 (DE)_3_-carrying plasmids pGro7/GroEL and pET22b *Sso*Pox-W263I for chaperone and *Sso*Pox-W263I expressions were used^30^. A first preculture of 100 mL was made from glycerol stock in LB medium (supplemented with 100 mg.L^−1^ ampicillin and 34 mg.L^−1^ chloramphenicol) over 4 h (OD_600_ = 2) at 37 °C with stirring at 140 rpm. A second preculture of 5.2 L was inoculated from the previous one in the same medium at 37 °C over 4 h (OD_600_ = 2) with stirring at 180 rpm. Five liters of preculture was used to inoculate 495 L of ZYP-5052 medium complemented with ampicillin (100 mg.L^−1^) and chloramphenicol (34 mg.L^−1^)^59^. The culture was performed at 37 °C with pO_2_ of 20%, aeration of 100–500 slpm (standard liter per minute) and stirring at 60–160 rpm. When the OD_600_ reached 0.8–1, protein production was induced by decreasing the temperature to 23 °C with addition of CoCl_2_ (200 μM) and L( + )-arabinose (0.2% w/v). After 12 h, cells were harvested by centrifugation at 11,000 g (clarifier Clara 15 Alfa Laval, flow rate 1 L.min^−1^). The cell pellet was resuspended in lysis buffer (50 mM HEPES, 150 mM NaCl, 0.2 mM CoCl_2_, 10 mg.L^−1^ DNase I, 250 mg.L^−1^ Lysosyme, 0.1 mM PMSF and pH = 8.0) and frozen at −80 °C for 48 h. The extract was then sonicated (Sonifier 450 with 902 R needle, Branson) and centrifuged for 5 min at 4 °C and 18,000 g. The supernatant was heated 30 min at 80 °C followed by another centrifugation at 18,000 g and 4 °C for 25 min to remove precipitate. Supernatant was ultrafiltrated (PES 10 kDa, Synder) and atomized (Mini Spray dryer B290, Buchi). Unless otherwise specified, 1.5 U of *Sso*Pox-W263I in 0.5 mL were used for the experiments.

### Paraoxonase activity measurement

The phosphotriesterase activity was measured with ethyl paraoxon (Sigma Aldrich) as previously described^48^. Briefly, in a 96-well plate, 2 μL of enzymatic solution were added to 98 μL of activity buffer (HEPES 50 mM, NaCl 150 mM and pH = 8.0). 100 μL of 2 mM ethyl paraoxon in activity buffer were added to each well for starting the reaction and the OD_405nm_ was followed with a microplate reader (Synergy HT, BioTek, USA) for 10 min.

### Heat shock resistance

The heat shock was performed using a dry bath (Isotemp, Fisher Scientific). Solid (i.e. atomized) or liquid (i.e. atomized enzyme resuspended in ultrapure water) enzyme was aliquoted in 1.5 mL Eppendorf tubes. The temperature was set from 40 °C to 120 °C for the resuspended samples and from 40 °C to 150 °C for the powder samples during 5 min. After the heat shock, the samples were cooled 5 min at ambient temperature (23 °C) and then, for the powder samples, resuspended in ultrapure water. As control, samples were left at ambient temperature (23 °C).

### Tolerance to solvents

The test was performed using atomized enzyme aliquoted in 3 mL glass vials (Wheaton, USA). 16 solvents were used: acetone, acetonitrile, butanone, butyl acetate, chloroform, dichloromethane, diethyl ether, ethanol, ethyl acetate, isopropanol, methanol, methoxypropanol, methylcyclohexane, petroleum ether, toluene, xylene (all purchased from Sigma Aldrich). To vials containing 1.5 U of *Sso*Pox-W263I, 500 μL of pure solvent was added and was followed by 30 s stirring with a vortex. Vials were let open under a chemical hood for 1 h 50 min followed by a 10–15 min evaporation under a nitrogen gas flux. Finally, the dry residue obtained was resuspended in 500 μL ultrapure water. As control, samples directly resuspended in water with a 2 h evaporation under a nitrogen gas flux (group used for relative activity calculation) and without. The last condition was used to check the impact of evaporation on *Sso*Pox-W263I activity.

### Reactivation by culture supernatants

The reactivation was investigated with four bacterial strains: *Pseudomonas aeruginosa* PAO1*, Acinetobacter baumannii* AYE*, Bacillus cereus* CIP6624T (clinical strain), and *Staphylococcus aureus* ATCC 29213. Strains were grown on 5% sheep blood Columbia agar plate and incubated at 37 °C overnight. From one colony, 15 mL LB medium was inoculated and incubated 24 h at 37 °C with stirring (600 rpm). The culture was then centrifuged at 8,000 g for 10 min to pellet down bacterial cells. The supernatants were collected and filtered over a 0.22 μm-filter. Aliquots of filtered supernatants were heated in a dry bath at 100 °C for 10 min to remove the majority of thermolabile compounds.

Unheated supernatants were used to resuspend solid enzyme heated or not at 150 °C for 5 min in a dry bath. Heated enzyme was also resuspended in heated supernatants. The samples were incubated at room temperature (23 °C) for 1 h. As control, the enzyme was resuspended in sterile LB medium. Furthermore, the potential activity on paraoxon of LB, with supernatants heated or not, was controlled using 2 μL and the resulting value was subtracted to each category of samples.

### Statistical analyses

Statistical analyses were performed using SPSS v22 software. The type I error, or α, was set at 0.05. First, the Shapiro-Wilk’s test and the Levene’s test were used in order to check the normality and equality of variance assumptions for each group containing LB controls and supernatant treated samples. One-way ANOVA was then performed on each group. In case of unequal variance, the Welch’s Test and Brown-Forsythe’s test were used to confirm the significant difference observed with the one-way ANOVA. Five orthogonal contrasts were analyzed to evaluate the significance of the results. Following the Levene’s test results, the p-value of contrasts was calculated considering the equivalence or lack of equivalence of variances. Furthermore, according to the Bonferroni correction, the p-value of each contrast was compared to α = 0.01 to give a global type I error of 0.05. In total, five contrasts comparing two conditions were used: A versus B, A versus C, B versus D, B versus E and D versus E.

### Sterilization processes

Autoclaving was performed in a 50-mL glass bottle (Duran, Germany) with 60 U of *Sso*Pox-W263I in powder directly or resuspended in 20 mL of ultrapure water. The autoclaving cycle was adapted to small volume in order to have 15 min at 121 °C with a quick temperature increase and decrease. After samples were cooled down, 20 mL of water were added to the autoclaved powder sample. As control, a freshly made 3 U/mL (equivalent to 1 mg of enzyme/mL) water solution was used.

β-radiation sterilization was performed in 3 mL glass vials on 12 U of *Sso*Pox-W263I in powder directly or resuspended in 1 mL of ultrapure water. The samples were put under an electron beam (frequency of 520 Hz) 2.6 mm wide with a speed of 0.85 m.min^−1^. One, two and four passages through the beam were enough to apply a dose of 25, 50 and 100 kGy. As control, samples without β-radiation treatment but with the same environmental conditions were used.

Ethylene oxide sterilization was performed on 12 U of *Sso*Pox-W263I in powder contained in flat pouches (SPS, France). The samples were submitted to from one to three consecutive cycles of sterilization by ethylene oxide. Briefly, a cycle consisted of a preheating time (from 51 to 67 min) followed by a conditioning time (longer than 180 min) at the end of which, the temperature was between 40–50 °C and humidity longer than 50%. The sample was then exposed to ethylene oxide for 300–310 min with a temperature of 40–50 °C and a weight of gas of 10.7–13.0 kg. As control, samples without treatment were used.

### Room temperature storage

To maintain an anhydrous atmosphere, 1.5 mL Eppendorf tubes containing enzyme powder were stored with desiccant packets (Clariant, Switzerland). Every week aliquots were resuspended in 500 μL of ultrapure water and the activity was measured. The results were normalized as compared to the control at day 0.

### Low temperature kinetics

The reaction was performed in activity buffer complemented with 50% of glycerol. Two separated solutions were prepared containing either 500 μM of ethyl paraoxon or 0.06 U/mL of *Sso*Pox-W263I. Both solutions were cooled to −18 °C. In a 2 mL Eppendorf tube at −18 °C, 1 mL of each were mixed together to start the reaction. Every hour, 100 μL was removed and mixed with 100 μL of chloroform and vortexed. The organic phase was transferred into gas-chromatography vial. The same experiment was performed at room temperature as control.

GC analysis were performed as follow: 100 μL of reaction medium was extracted with 100 μL of chloroform. Organic extracts were analyzed by using a Clarus 500 gas chromatograph equipped with a SQ8S MS detector (Perkin Elmer, Courtaboeuf, France). 1 μL of organic extract was volatilized at 220 °C (split 15 mL/min) in a deactivated FocusLiner with quartz wool (SGE, Ringwood, Australia) and compounds separated on an Elite-5MS column (30 m, 0.25 mm i.d., 0.25 mm film thickness) during 12 minutes using a temperature gradient (80–280 °C at 30 °C/min, 5 minutes hold). Helium flowing at 2 mL/min was used as carrier gas. The MS inlet line was set at 280 °C and electron ionization source at 280 °C and 70 eV. Full scan monitoring was performed from 40 to 400 m/z in order to identify chemicals by spectral database search using MS Search 2.0 operated with the Standard Reference Database 1 A (National Institute of Standards and Technology, Gaithersburg, MD, USA). Selected Ion Recording using base peaks ions was applied in order to specifically monitor pesticides and collect peak areas for kinetics. Peak areas were converted to percentage of initial concentration value. All samples were analyzed in short periods of time to avoid signal drift. All data were processed using Turbomass 6.1 (Perkin Elmer).

It was reasonably assumed that the substrate concentration was negligible as compared to K_M_. k_cat_/K_M_ was thus estimated by using the one phase decay function in GraphPad Prism v6. Curves were then fitted using One-Phase Decay non-linear regression with the equation:


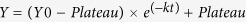


Where *Y*0 = 0% and *Plateau* = 100%. The resulting rate constant K was divided by the enzyme molar concentration to estimate the k_cat_/K_M_ in M^−1^.s^−1^.

Temperature dependence of pH for HEPES was previously described with a ΔpKa value of −0.14 every 10 °C^60^. So by varying the temperature from −18 °C up to 70 °C, pH may vary within a range of 1.5 unit. However, the tolerance of *Sso*Pox to pH changes was previously reported and no significant modification of the specific activity was observed from pH 7.0 to 9.0^14^.

### Immobilization

The immobilization was performed with 15 U/mL of enzyme mixed in a 3% (w/v) solution of sodium alginate. Glutaraldehyde was added to the solution at a final concentration of 0.5%. Alginate beads were obtained by dropping the enzymatic solution with a 1 mL syringe connected to a 35 g needle, in 40 mL of 0.2 M CaCl_2_ solution. After 1 h with stirring at room temperature, the beads were washed for 30 min with 30 mL of the activity buffer complemented with 0.2 M CaCl_2_.

Activity of the beads was evaluated in 5 mL solution of 1 mM ethyl in activity buffer complemented with 0.2 M CaCl_2_. After 3 min of reaction with upside down agitation, 200 μL of the solution was transferred into a 96-well plate and the OD_405nm_ was followed for 10 min. For the recycling test, after each cycle of catalysis, the beads were washed twice for 10 min with 30 mL of CaCl_2_ complemented activity buffer. As negative control, empty beads were made and no activity was detected. For yield determination, a positive control was made with 15 U of free enzyme resuspended in 1 mL of water and diluted to 1/10 with complemented activity buffer. Afterward, 1 mL of the diluted solution was added to 4 mL of ethyl paraoxon solution (final concentration 1 mM).

## Additional Information

**How to cite this article**: Rémy, B. *et al*. Harnessing hyperthermostable lactonase from *Sulfolobus solfataricus* for biotechnological applications. *Sci. Rep.* **6**, 37780; doi: 10.1038/srep37780 (2016).

**Publisher's note:** Springer Nature remains neutral with regard to jurisdictional claims in published maps and institutional affiliations.

## Figures and Tables

**Table 1:** Catalytic efficiencies of *Sso*Pox-W263I with paraoxon at different temperatures.

Temperature	Reaction conditions	*k*_cat_/K_M_ (M^−1^.s^−1^)*
−18 °C	Activity buffer + 50% glycerol	1.4 × 10^3^
23 °C	Activity buffer	1.2 × 10^3^**
23 °C	Activity buffer + 50% glycerol	9.7 × 10^3^
70 °C	Activity buffer	2.5 × 10^4^
70 °C	Activity buffer + 50% glycerol	2.0 × 10^4^

^*^k_cat_/K_M_ were estimated for a concentration of 250 μM of paraoxon. It was assumed that the substrate concentration was negligible as compared to K_M_. *k*_cat_/K_M_ was thus estimated by using the one phase decay function in GraphPad Prism v6. The resulting rate constant k was divided by the enzyme molar concentration to estimate the *k*_cat_/K_M_ in M^−1^.s^−1^.

^**^Data taken from^48^.
